# Regulation of flavonol content and composition in (Syrah×Pinot Noir) mature grapes: integration of transcriptional profiling and metabolic quantitative trait locus analyses

**DOI:** 10.1093/jxb/erv243

**Published:** 2015-06-12

**Authors:** Giulia Malacarne, Laura Costantini, Emanuela Coller, Juri Battilana, Riccardo Velasco, Urska Vrhovsek, Maria Stella Grando, Claudio Moser

**Affiliations:** ^1^Genomics and Biology of Fruit Crops Department, Research and Innovation Centre, Fondazione Edmund Mach, Via E. Mach 1, 38010 S. Michele all’Adige, Trento, Italy; ^2^Computational Biology Department, Research and Innovation Centre, Fondazione Edmund Mach, Via E. Mach 1, 38010 S. Michele all’Adige, Trento, Italy; ^3^Food Quality and Nutrition Department, Research and Innovation Centre, Fondazione Edmund Mach, Via E. Mach 1, 38010 S. Michele all’Adige, Trento, Italy

**Keywords:** Berry, flavonols, candidate gene, metabolic profiling, microarray, quantitative trait loci, segregating population, *Vitis vinifera.*

## Abstract

Flavonols are a ubiquitous class of flavonoids that accumulate preferentially in flowers and mature berries. Besides their photo-protective function, they play a fundamental role during winemaking, stabilizing the colour by co-pigmentation with anthocyanins and contributing to organoleptic characteristics. Although the general flavonol pathway has been genetically and biochemically elucidated, the genetic control of flavonol content and composition at harvest is still not clear. To this purpose, the grapes of 170 segregating F_1_ individuals from a ‘Syrah’×’Pinot Noir’ population were evaluated at the mature stage for the content of six flavonol aglycons in four seasons. Metabolic data in combination with genetic data enabled the identification of 16 mQTLs (metabolic quantitative trait loci). For the first time, major genetic control by the linkage group 2 (LG 2)/*MYBA* region on flavonol variation, in particular of tri-hydroxylated flavonols, is demonstrated. Moreover, seven regions specifically associated with the fine control of flavonol biosynthesis are identified. Gene expression profiling of two groups of individuals significantly divergent for their skin flavonol content identified a large set of differentially modulated transcripts. Among these, the transcripts coding for MYB and bZIP transcription factors, methyltranferases, and glucosyltranferases specific for flavonols, proteins, and factors belonging to the UV-B signalling pathway and co-localizing with the QTL regions are proposed as candidate genes for the fine regulation of flavonol content and composition in mature grapes.

## Highlight

Novel candidate genes for the fine regulation of flavonol content in ripe berries are identified through integration of transcriptional profiling and metabolic QTL analyses of a segregating grapevine progeny.

## Introduction

Flavonols in grapes, as in almost all higher plants, are an important class of bioactive compounds. In grapes they are most abundant in the flowers and skins of the berries, although they have also been reported in leaves and stems (Hmamouchi *et al.*, 1996). They are produced by the flavonoid biosynthetic pathway, which also gives rise to anthocyanins and condensed tannins. Among flavonoids, they represent one of the most important classes in terms of concentration, especially in white grapes (Flamini *et al.*, 2013).

From an agronomic perspective, flavonols are important phytochemicals for viticulture and enology. They stabilize the colour of red wines, forming molecular complexes with anthocyanins, a phenomenon known as co-pigmentation (Boulton, 2001). In addition, they confer antioxidant properties to the wine (Yang *et al.*, 2009), which translates into health benefits for the consumer (Nassiri-Asl and Hosseinzadeh, 2009).

From a taxonomic perspective, flavonols are useful markers since their profiles vary considerably among grapevine cultivars, ultimately contributing to wine typicity, and, as such, they have been used for the authentication and the varietal differentiation of grapes and wine (Mattivi *et al.*, 2006; Castillo-Munoz *et al.*, 2007, 2010).

Flavonols possess a 3-hydroxyflavone backbone and differ in the number and type of substitutions on the B ring. They usually occur as 3-*O*-glycoside derivatives with the sugar attached to the 3′ position of the flavonoid skeleton. Although the presence of up to six different aglycons is expected in grape berry skin (Fujita *et al.*, 2006), the lack of expression of the flavonoid 3′,5′-hydroxylase genes restricts them to the mono- and di-substituted derivatives kaempferol (Kaemp), quercetin (Que), and isorhamnetin (Isor) in white grapes. In contrast, red grapes usually also accumulate the tri-substituted flavonols myricetin (Myr), laricitrin (Lar), and syringetin (Syr) (Castillo-Munoz *et al.*, 2007, 2010).

Flavonols accumulate exclusively in the outer epidermis of the grape berry pericarp and in some layers of the seed coat (Fontes *et al.*, 2011). In particular, they are stored in the inner, thick-walled layers of hypodermis, and the thickness of the berry skin influences their concentration (McDonald *et al.*, 1998). The localization of flavonols is related to their role as protectants against UV, extreme temperatures, as well as free radicals (Winkel-Shirley, 2002). As a consequence, synthesis of flavonols is a light-dependent process regulated by light-dependent modulation of the main genes involved in their biosynthesis (Czemmel *et al.*, 2009). It was recently demonstrated that UV-B light has a greater effect on mono- and di-substituted flavonol accumulation through the up-regulation of flavonoid 3′-hydroxylase genes (Martinez-Luscher *et al.*, 2014).

During development, flavonol biosynthesis begins in the flower buttons, decreases between flowering and berry set, and then reaches a peak at 3–4 weeks post-véraison (Downey *et al.*, 2003).

In general, the total amount of flavonols varies extensively among *Vitis vinifera* varieties, ranging from 1mg kg^–1^ to 80mg kg^–1^ of fresh berry weight, with red cultivars usually being richer than white ones (Mattivi *et al.*, 2006; Castillo-Munoz *et al.*, 2007, 2010), and reaching >100mg kg^–1^ in some wild *Vitis* species (Liang *et al.*, 2012). The level and the distribution of the different classes of flavonols can be strongly affected by agronomic and environmental conditions which impact sunlight exposure and water status. However, there is evidence that flavonol profiles, as in general phenolic profiles, are also highly influenced by genetic factors playing a role at the cultivar level (for reviews, see Flamini *et al.*, 2013; Teixeira *et al.*, 2013).

The general flavonol biosynthetic pathway has been genetically and biochemically elucidated in many plant species, and recently it was also described in grapevine: their synthesis is catalysed by the action of ﬂavonol synthase (VvFLS), an enzyme which uses di-hydroﬂavonols as substrates and whose encoding gene is temporally and specifically regulated by a R2R3-MYB transcription factor, named VvMYBF1 (Downey *et al.*, 2003; Czemmel *et al.*, 2009).

Also determining steps in flavonol accumulation are reactions driven by chalcone isomerase (VvCHI) and by ﬂavonoid 3′-hydroxylase (VvF3′H) and ﬂavonoid 3′,5′-hydroxylase (VvF3′5′H), which mediate the addition of hydroxyl groups to the B-ring of ﬂavanones, ﬂavones, and dihydroﬂavonols (Jeong *et al.*, 2006). In particular, VvF3′5′H competes with VvF3′H and VvFLS for dihydroﬂavonol substrates and, therefore, changes in any of these enzymatic activities by external factors, such as an increase of F3′H and FLS activities by UV-B, may affect the flavonol profile in favour of mono- and di-substituted derivatives (Martinez-Luscher *et al.*, 2014). Subsequently, glycosylation of ﬂavonols driven by the glucosyltranferases 5 (VvGT5) and 6 (VvGT6) enhances their water solubility, allowing the accumulation of high concentrations of these compounds (Ono *et al.*, 2010). Despite this knowledge, understanding of the fine control of flavonol biosynthesis requires further investigation, especially at the stage of technological maturity of the grapes, the most relevant for industry.

In this study, advantage was taken of a segregating population derived from a cross between two *V. vinifera* cultivars with significantly divergent flavonol content in the skin at the mature berry stage (‘Syrah’×’Pinot Noir’). The progeny were characterized in four seasons by analysing the flavonol content and composition. Overall, 22 traits were considered for analysis using two parallel approaches: (i) individuals of the population exhibiting the extremes of flavonol content were analysed at the transcriptional level using microarrays; and (ii) the metabolic data were used in combination with a genetic map (Costantini *et al.*, 2015) for quantitative trait locus (QTL) analysis. This enabled the identification and characterization of seven mQTLs exclusively associated with flavonol biosynthesis. The study revealed a large effect QTL controlling all the traits under analysis which consistently co-localized with the major locus for anthocyanin berry content on linkage group 2 (LG 2; Costantini *et al.*, 2015). The identification of a common genetic control was completely novel. By integrating the results of the two approaches, a list of candidate genes for the regulation of flavonol content and composition in mature grapes was produced based on co-localization of significantly modulated transcripts with metabolic QTLs (mQTLs) and on available knowledge on flavonol metabolism. The identification of new genetic determinants of flavonol varietal composition at technological maturity represents a valuable result for the wine growing industry.

## Materials and methods

### Plant material and sampling

The content of flavonols in the berry skin was evaluated in 170 F_1_ individuals derived from the cross ‘Syrah’×’Pinot Noir’ (clone ENTAV 115) and in the parental lines. All plants were grown at the Giaroni experimental field of FEM (Edmund Mach Foundation, San Michele all’Adige, Italy, 46°18′N, 11°13′E). Plants were grafted on the rootstock Kober 5BB and trained according to the Guyot system. Two clusters of the same plant were sampled for each genotype at technological maturity (18 °Brix) in four different seasons (2007–2008–2009–2011) and stored at –80 °C until use. Anthocyanin content was analysed on the same samples in a parallel study (Costantini *et al.*, 2015). After ranking the median value obtained considering the concentration of all the compounds in all the seasons, eight individuals of the cross were divided into two groups significantly divergent for their content (HFPs, high flavonol producers, codes 16, 56, 63, 223; and LFPs, low flavonol producers, codes 64, 256, 260, Pinot Noir) and subjected to gene expression profiling. The selection of the individuals was statistically supported by analysis of variance (ANOVA) followed by Tukey HSD test (*P*-value <0.05) using Statistica 9 software (StatSoft, Tulsa, OK, USA). For microarray analysis, a representative sample of 50 berry skins was collected from each of the eight individuals (during the 2007 season) at three berry developmental stages: hard green berry (33E-L, PV), véraison (35E-L, 50% coloured berries, VER), and maturity (38E-L, 18 °Brix, MAT). For real-time reverse transcription–PCR (RT–PCR) analysis, the same samples used for microarray analysis were used for technical validation, while a representative sample of 50 berry skins was collected from three biological replicates of the eight individuals (during the 2011 season) at the same three developmental stages for the assessment of the biological variability in the gene expression.

### HPLC-DAD-MS analysis of flavonols

For each genotype of the population, 20 berries representative of the two collected clusters were considered for flavonol analysis. Flavonol extraction, acid hydrolysis of flavonol glycosides, and high-performance liquid chromatography-diode array coupled to mass spectrometer detector (HPLC-DAD-MS) analysis were performed as previously described (Mattivi *et al.*, 2006). Each flavonol was identified by comparison of retention time, UV spectra at 370nm, and MS spectra in positive mode with those of the corresponding standards. The content was quantified in mg kg^–1^ of fresh berry weight by means of the external standard method, specific for each compound. Values under the limit of quantification (LOQ) were imposed equal to zero in the final quantification: Myr LOQ=0.66mg l^–1^, Que LOQ=0.46mg l^–1^, Lar LOQ=0.55mg l^–1^, Kaemp LOQ=0.51mg l^–1^, Isor LOQ=0.63mg l^–1^, Syr LOQ=0.54mg l^–1^.

Statistical analyses of biochemical data were performed with the software SPSS 11.0 (IBM SPSS Statistics). The normality of trait distribution was checked by a Kolmogorov–Smirnov test. If necessary, data were log-transformed with a ln(1+*x*) function. Correlations between traits within years and between years within traits were determined using the non-parametric Spearman correlation coefficient.

### QTL analysis

For QTL identification, a genetic linkage map based on the segregation of 690 simple sequence repeat (SSR) and single nucleotide polymorphism (SNP) markers in 170 F_1_ individuals (Costantini *et al.*, 2015) was used. QTL analysis was performed using MapQTL 6.0 (Van Ooijen, 2009) with simple interval mapping and multiple QTL mapping functions, adopting the same procedure as in Costantini *et al.* (2015). As a general trend, QTLs for the different flavonols were identified by analysing the whole progeny, while QTLs for flavonol ratios were identified by considering the coloured progeny only. In contrast, in the case of Kaemp, Que, and Isor, the analysis was run both in the whole and in the white progeny, to test if the co-existence of anthocyanins together with flavonols in the mature berry skin of coloured individuals could affect the regulation of flavonol biosynthesis. Suffixes (a and b) were adopted to indicate different regions on the same LG where QTLs were found for flavonol (this work) and anthocyanin (Costantini *et al.*, 2015) content.

The 12× grape reference genome (http://genomes.cribi.unipd.it/) was used to extract version 1 of the gene predictions underlying QTLs. A Fisher’s exact test was applied to evaluate the over-representation of specific functional categories in the QTL regions as in Costantini *et al.* (2015). The combination of all the regions controlling a given trait was tested. Adjusted *P*-values obtained applying the Benjamini–Hochberg method (Benjamini and Hochberg, 1995) for multiple testing correction were considered significant when ≤0.05.

### RNA isolation, microarray hybridization, and data analysis

RNA isolation, microarray hybridization, and data analysis were carried out as described in detail at the GEO database under the accession GSE42909 (http://www.ncbi.nlm.nih.gov/geo/query/acc.cgi?acc=GSE42909 accessed 19 December 2014). Briefly, a total of 24 hybridizations were performed (eight F_1_ individuals×three developmental stages) on the custom Affymetrix GrapeGen GeneChip^®^, representing ~18, 711 unique genes. Information about the GeneChip^®^ is available in ArrayExpress (Pontin *et al.*, 2010) and GEO (Ali *et al.*, 2011) under the accession numbers E-MEXP-2541 and GSE24561, respectively. The Robust Multi-array Analysis (RMA) algorithm was used for background correction, normalization, and expression level summarization (Irizarry *et al.*, 2003). Probe sets with significant differential expression (DEPs) between LFPs and HFPs were selected in two steps and under the assumption that the four individuals of the LFP and HFP groups can be treated as biological replicates (pseudoreplicates). Step 1: within each group (LFPs and HFPs), the data were analysed through pairwise comparisons of developmental stages (PV versus VER, PV versus MAT, and VER versus MAT). Differential expression analysis was performed with the Bayes *t*-statistics from LIMMA (Linear Models for Microarray Data), and *P*-values were corrected for multiple testing using the Benjamini–Hochberg method (Benjamini and Hochberg, 1995). Three lists of DEPs for each group were thus obtained [with a false discovery rate (FDR) <5% and a cut-off of 2-fold change (FC) between each pair of stages]. Step 2: the lists of DEPs were then compared between LFPs and HFPs to provide a final list of probe sets either exclusively modulated in LFPs or in HFPs, or modulated in both groups but with low correlation [−0.5>(logFC_LFPs_–logFC_HFPs_)_pairwise comparison_>0.5]. The two-step analysis was preferred to the direct comparison between LFPs and HFPs at each developmental stage, to reduce the number of false DEPs caused by a non-perfect phasing of the samples of the two groups.

Probe set annotation was updated according to the 12Xv1 prediction (http://genomes.cribi.unipd.it/ accessed 19 December 2014), and functional categorization was inferred from Lijavetzky *et al.* (2012). In particular, each probe set was assigned to one of 516 categories of a custom-made catalogue based on plant-related terms from the GO vocabulary and MIPS FunCat (Ashburner *et al.*, 2000; Ruepp *et al.*, 2004). Therefore, hypergeometric tests (*P*<0.05) were applied to evaluate the enrichment of a functional category of the third level among the DEPs, in comparison with the distribution of the 23, 096 GrapeGen probe sets as calculated from Lijavetzky *et al.* (2012).

### Quantitative RT-PCR analysis

Total RNA from all the samples of the eight individuals collected in both the 2007 (also used in microarray experiments) and 2011 seasons was treated with DNase I (Ambion, Life Technologies), and the first-strand cDNA was synthesized from 1.5 μg of treated total RNA using Vilo™ reverse transcriptase (Life Technologies Ltd, Paisley, UK). Reactions were carried out with Platinum SYBR Green qPCR SuperMix-UDG (Life Technologies Ltd) and specific primers (details are given in Supplementary Table S5 available at *JXB* online) using a ViiA7 real-time PCR machine (Applied Biosystems, Foster City, CA, USA). Plates were set up according to the sample maximization strategy proposed in Hellemans *et al.* (2007). Each sample was examined in three technical replicates, and dissociation curves were analysed to verify the specificity of each amplification reaction. The conditions of PCRs and the protocol of analysis were the same as reported in Giacomelli *et al.* (2012). Non-baseline-corrected data were imported in LinReg software to calculate reaction efficiencies (Ruijter *et al.*, 2009). Six housekeeping genes (*EF1α*, *GAPDH*, *ACTIN*, *UBIQUITIN*, *SAND*, and *α-TUBULIN*; Supplementary Table S5) were tested for their stability in each experiment using the GeNorm software (Vandesompele *et al.*, 2002). Normalized relative quantities (NRQs) were then calculated by dividing the RQ by a normalization factor, based on the expression of the two most stable reference genes (*GAPDH* and *ACTIN*) (Reid *et al.*, 2006).

### Candidate gene selection

Candidate genes were selected among the 12Xv1 predictions included in the confidence interval of reliable QTLs in the presence of at least one of the following functional pieces of evidence: (i) involvement in trait regulation based on the literature; (ii) differential expression between LFPs and HFPs (Supplementary Table S4 at *JXB* online); (iii) expression profile consistent with the accumulation of flavonols during berry development; in particular it was considered that Kaemp and Que show the highest peak of accumulation at flowering, and Isor and tri-hydroxylated flavonols at maturity (Sternad Lemut *et al.*, 2013); expression in berry skin higher than in berry flesh (data from (Fasoli *et al.*, 2012; Lijavetzky *et al.*, 2012; Gouthu *et al.*, 2014); (iv) co-expression with genes involved in the regulation of flavonol biosynthesis or in flavonoid metabolism [data from MarcoPaolo (provisional link, http://vitis.colombos.fmach.it/ accessed 19 December 2014), a grapevine compendium built based on COLOMBOS technology (Meysman *et al.*, 2014), and from the VTC database (Wong *et al.*, 2013); co-expression analyses by COLOMBOS were essentially based on contrast relevance and gene similarity scores, as described in depth in Supplementary Text S1, from Engelen *et al.* (2011); in both databases, the microarray and RNA-seq experiments related to berry and berry development were considered as data sets; and (v) assignment to functional categories significantly over-represented in QTL regions (Supplementary Table S6).

## Results and Discussion

The population obtained by crossing the two red-skinned varieties, Syrah and Pinot Noir, comprises individuals with white (25%) and coloured (75%) berries in agreement with a 1:3 segregation of a major locus and with the bi-modal distribution of white-to-coloured grapes for anthocyanin content as reported by Costantini *et al.* (2015).

**Fig. 1.**
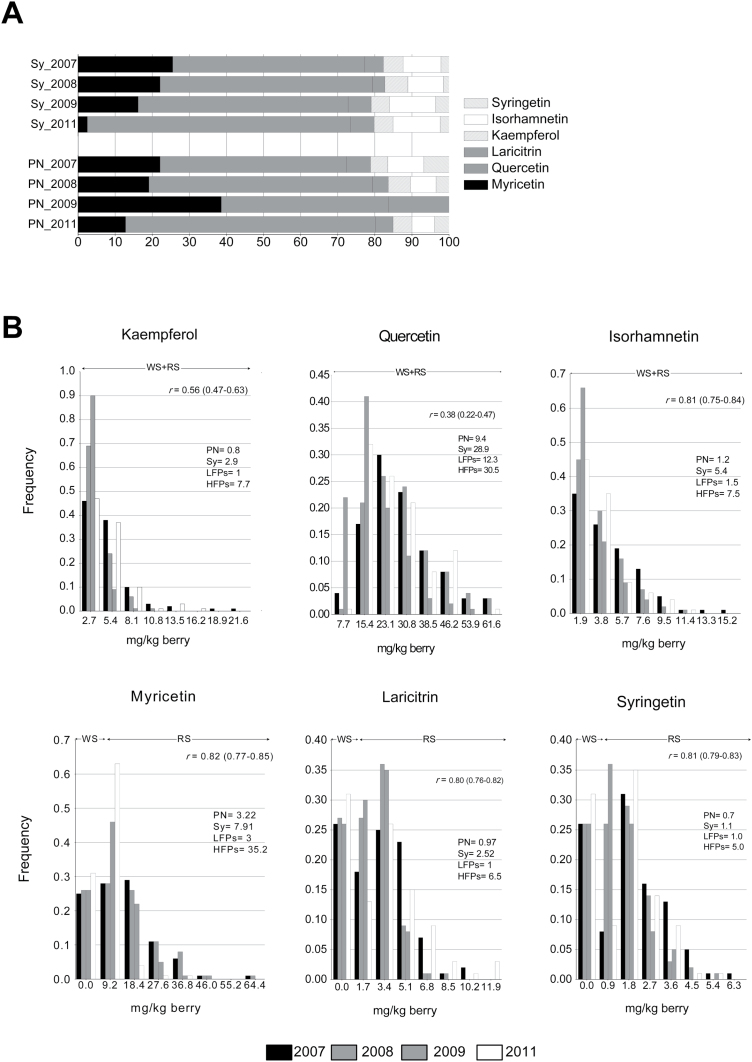
Variation of the flavonol content in the parental lines (A) and in the ‘Syrah’x‘Pinot Noir’ progeny (B) in berry skins at mature stage. (A) Profile of each flavonol in each year in the two parents of the cross. (B) Distribution of the progeny in classes at increasing content of each flavonol (mg kg^–1^ berry). In the right upper part of each plot, the Spearman rank-order correlation between years (*r*) (mean value and range of variation in parentheses) is shown for the whole progeny. Correlations are significant at the 0.01 level. For the two parents and for high- and low-flavonol producers, averaged values across years and across genotypes (in the second case) are reported. Abbreviations: Sy, Syrah; PN, Pinot Noir; LFPs, low-flavonol producers; HFPs, high-flavonol producers; WS, white-skinned individuals; RS, red-skinned individuals.

The total flavonol content in the four seasons was on average three times higher in ‘Syrah’ than in ‘Pinot Noir’, ranging from 34mg kg^–1^ to 57mg kg^–1^ for ‘Syrah’ and from 5mg kg^–1^ to 23mg kg^–1^ for ‘Pinot Noir’. In particular, Que and Myr were the most abundant flavonols in all the seasons, with Que being on average three and four times higher than Myr in ‘Pinot Noir’ and ‘Syrah’, respectively (Fig. 1A). The profiles among years were relatively stable in both cultivars, with few exceptions: in 2009, Kaemp, Isor, and Lar were completely undetectable in ‘Pinot Noir’, possibly compensated by an increase in Myr, with the latter being very low in ‘Syrah’ in 2011 (Fig. 1A). Moreover, looking at the ratios between tri-hydroxylated and di-hydroxylated flavonols (triOH/diOH), between 3′-methylated and 3′-hydroxylated flavonols (3′Meth/3′OH), and between 3′,5′-methylated and 3′,5′-hydroxylated flavonols (5′Meth/5′OH), it appears that they were on average higher in ‘Syrah’ than in ‘Pinot Noir’.

Considering the progeny, all six flavonol aglycons could be detected in almost all the red-skinned individuals, while the tri-hydroxylated flavonols (Myr, Syr, and Lar) were under the detection limit in the white-skinned progeny. The total flavonol content varied a lot in the progeny, ranging from 1mg kg^–1^ to 76mg kg^–1^ in the white-skinned offspring, and from 5mg kg^–1^ to 142mg kg^–1^ in the red-skinned individuals (Supplementary Table S1 at *JXB* online). These intervals are consistent with the reported range of variability of *V. vinifera* varieties: from 1mg kg^–1^ to 132mg kg^–1^ for white cultivars, and from 4mg kg^–1^ to 176mg kg^–1^ for red cultivars (Mattivi *et al.*, 2006; Liang *et al.*, 2011). The di-hydroxylated flavonol Que represented on average >70% of total flavonols in the white progeny, while Que and Myr together represented on average 80% of total flavonols in the coloured progeny, as previously observed (Mattivi *et al.*, 2006; Castillo-Munoz *et al.*, 2007, 2010). The Kaemp concentration was much higher in the white (max value=21mg kg^–1^ of fresh berry in 2007) than in the coloured progeny (max value=8mg kg^–1^ of fresh berry in 2011). In contrast, Isor was much higher in the coloured (max value=15mg kg^–1^ of fresh berry in 2007) than in the white progeny (max value=1mg kg^–1^ of fresh berry in 2011).

In all years, the content of the six flavonols in the berries of the whole progeny showed a continuous variation, although the distribution was specific for each single compound and year (Fig. 1B).

In addition, the progeny showed a transgressive segregation for all compounds in all vintages; that is, the two tails of the flavonol distributions were populated by individuals displaying a phenotype out of the range delimited by the parents. A significant number of offspring presented a content of each single flavonol much higher than ‘Syrah’, while ‘Pinot Noir’ displayed an average value of Kaemp, Lar, and Syr close to the minimum observed in the whole coloured progeny in all seasons (Fig. 1B).

The concentration of the different compounds was in general stable across years (correlation *r*>0.80, *P*<0.01, when considering the whole progeny), supporting a substantial genetic control of their biosynthesis. However, Kaemp and Que appeared more influenced than the others by the season (*r*=0.5 and *r*=0.4, respectively; Fig. 1B). This tendency might be linked in general to the effect of the variability of the environmental conditions among seasons and also to the developmental regulation of the different classes of flavonols.

Harvest is the time with the highest accumulation of tri-hydroxylated flavonols, matching well with the specific expression of flavonoid 3′,5′-hydroxylase genes (Jeong *et al.*, 2006); flowering, on the other hand, is the time when the ‘early peaking’ flavonols Kaemp and Que, as named by Sternad Lemut *et al.* (2013), have a peak of abundance (Fujita *et al.*, 2006).

Metabolite analysis has also clearly indicated that some flavonoid compounds are highly associated at the biosynthetic level. Indeed, high Spearman rank-order correlations (significance of 0.01) were found both within flavonols and between flavonols and anthocyanins (Supplementary Table S2 at *JXB* online), supporting a common enzymatic and/or transcriptional regulatory system, as previously suggested (Mattivi *et al.*, 2006; Martinez-Luscher *et al.*, 2014). This was so in the case of tri-substituted flavonols (*r*>0.7 between Lar and Syr, and Myr and Lar, and *r*>0.6 between Myr and Syr), di-substituted flavonols (*r*>0.7, between Que and Isor), and mono- and di-substituted flavonols (0.6<*r*<0.8). Furthermore, the analysis of the correlation between triOH/diOH, 3′Meth/3′OH, and 5′Meth/5′OH flavonols and anthocyanins suggests that some specific traits may be controlled by the same reaction(s). This could be the case of a common hydroxylase for the synthesis of Myr and delphinidin (*r*>0.6), or the same methyltransferase for the correlated 3′Meth/3′OH and 5′Meth/5′OH ratios (0.5<*r*<0.6). Conversely, the synthesis of Kaemp and diOH flavonols showed little or no correlation with that of anthocyanins and of triOH flavonols, as discussed above.

### QTL analysis

In this study, several QTLs for the content of each single flavonol, for the sum of and for the ratio between the concentrations of specific classes of flavonols have been identified.

When analysing the whole progeny, a major QTL for all the traits under analysis and for their sum (Tot_Flav) was identified on LG 2. In the case of Kaemp and Que, this QTL explained from 21% to 25% of phenotypic variance averaged over 3 and 2 years, respectively, and from 42% to 67% of variance averaged over 4 years in the case of Isor and triOH flavonols, respectively. The same QTL also regulates the ratio between specific classes of flavonols (explained variance from 15% to 35%). However, the position of the region on LG 2 associated with Kaemp and Que content was not stable over the different seasons (Supplementary Table S3 at *JXB* online).

It is noteworthy that the QTL on LG 2 coincides with the major locus controlling anthocyanin berry content (Costantini *et al.*, 2015) and co-localizes with a cluster of four *MYBA* genes, known to be essential for anthocyanin production via specific activation of the *UFGT* promoter (Kobayashi *et al.*, 2002). Although there was some indirect evidence that these two classes of flavonoids are at least partially connected (Mattivi *et al.*, 2006; Martinez-Luscher *et al.*, 2014) and there is a clear strict interdependency of all the classes belonging to flavonoid metabolism, this finding of a common genetic control was completely novel. Further support for this regulatory link comes from the observation that the ‘late peaking’ flavonols, namely Isor and the triOH compounds, which show an accumulation profile mimicking those of the anthocyanins, are those where a greater extent of the phenotypic variation is explained by the QTL on LG 2. To investigate further the genetic control of this chromosomal region on flavonol content, advantage was taken of the data on the white-skinned individuals where *MYBA* genes are not expressed, anthocyanins are absent, and Kaemp, Que, and Isor are the only flavonols detected. For these compounds the analysis was run considering both the data of the whole progeny and the data of the white-skinned individuals (Kaemp_white progeny, Que_white progeny, and Isor_white progeny), independently.

Interestingly, different regions (diverse from the QTL on LG 2) were found associated with the synthesis of Que, Isor, and their ratio, depending on the data set, while no QTL was found for Kaemp synthesis considering only the white-skinned individuals. A major QTL for Que_white progeny mapped to LG 10 (explaining 39% of the total variance in 2 years), while two different QTLs for Que in the whole progeny were detected on LG 2 and on LG 17 (explaining 25% and 11% of the total variance in 2 years). In the same way, a QTL for Isor_white progeny mapped to LG 11 (explaining 39% of the total variance, 3 years), while three different QTLs for Isor_whole progeny mapped to LG 2 (explaining 67% of the total variance) and to LGs 5 and 15 (explaining in both cases 5% of total variance). Finally, four different QTLs for 3′OMT_white progeny were identified on LGs 4a, 11b, 14, and 18b (explaining 33, 43, 22, and 19% of total variance, respectively). The regions associated with Isor/Que_whole progeny were completely different and mapped on LGs 1a, 15, 16, and 17 (explaining 25, 10, 15, and 13% of the total variance, respectively).

These results suggest that the genetic regulation of the synthesis of non-triOH flavonols may be different in white-skinned individuals compared with red-skinned ones, where the influence of the LG 2/*MYBA* region is predominant.

**Fig. 2.**
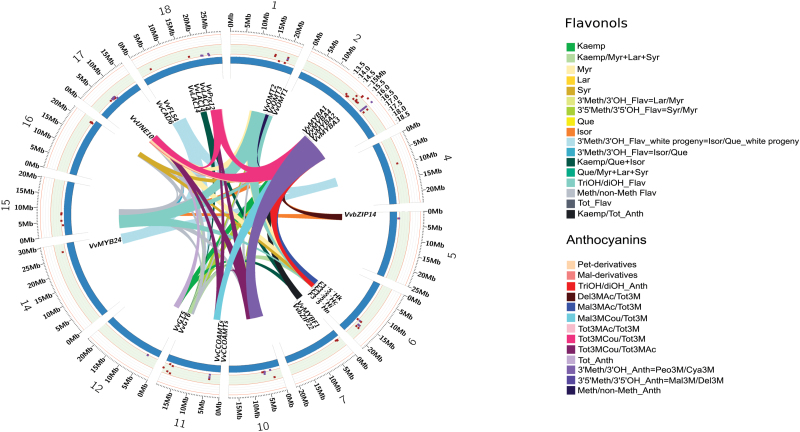
Plot of the chromosomal position of the identified QTLs for flavonol content and composition in the ‘Syrah’x‘Pinot Noir’ progeny. The regions identified by Costantini *et al.* (2015) controlling anthocyanin content are also presented. In both cases, only the traits regulated by at least two regions are visualized as coloured connections between chromosomes. External numbers and axes, for each blue arc, indicate the chromosomes and the physical length in megabases (Mbp), respectively. The concentric orange coloured circles represent three different levels of the explained variance for each trait (10, 50, and 90% outwards), while the red and violet squares correspond to flavonols and anthocyanins, respectively. A single colour code is associated with each trait as in the key. Gene names indicate the identified candidate genes to which particular attention was paid in the text.

Several other QTLs appeared relevant for controlling the berry flavonol content at maturity (Fig. 2; Table 1; Supplementary Table S3 at *JXB* online). Seven QTLs on LGs 4a, 7a, 11b, 14, 15, 16, and 18a were exclusively associated with the control of flavonol biosynthesis, while the others on LGs 1a, 5, 6, 10, 11a, 12a, 17, and 18b co-localize with regions associated with anthocyanin biosynthesis (Costantini *et al.*, 2015; Fig. 2).

**Table 1.** Genes with a potential role in the regulation of flavonol content and compositionOnly QTL regions exclusively associated with flavonol content are shown, while those associated with both flavonol and anthocyanin content are listed in Supplementary Table S7 at *JXB* online.

**Candidate gene**	**Functional evidence**	**This study**
**LG 4a: 27.7–36.5 cM = 6,815,958-11,887,467bp (192 gene predictions**)		
Harpin-induced protein: VIT_04s0069g01010*	Induced at maturity both in HFPs and LFPs (higher fold-change in HFPs); co-expressed with laccase and genes of the “phenylpropanoid catabolic process”; involved in the response to biotic stimulus.	✓
Glutathione S-transferase 26 GSTF12: VIT_04s0079g00690*	Induced at maturity both in HFPs and LFPs (slightly higher fold change in HFPs); higher expression in skin than in flesh (Lijavetzky *et al.*, 2012); expressed in the skin from véraison (Fasoli *et al.*, 2012); co-expressed with genes of the “quercetin O-glucoside metabolic process”.	✓
Cyclic nucleotide-regulated ion channel (CNGC14): VIT_04s0069g00790*	Induced at maturity both in HFPs and LFPs; higher expression in skin than in flesh (Lijavetzky *et al.*, 2012); co-expressed with genes of the “flavonoid metabolic process”; involved in the response to biotic stimulus.	✓
Glutamate receptor protein: VIT_04s0069g00530, VIT_04s0069g00710, VIT_04s0069g00330, VIT_04s0069g00540, VIT_04s0069g00610, VIT_04s0069g00010, VIT_04s0069g00060, VIT_04s0069g00170, VIT_04s0069g00200, VIT_04s0069g00210, VIT_04s0069g00230, VIT_04s0069g00260, VIT_04s0069g00270, VIT_04s0069g00290, VIT_04s0069g00380, VIT_04s0069g00390, VIT_04s0069g00550, VIT_04s0069g00560, VIT_04s0069g00620, VIT_04s0069g00630, VIT_04s0069g00670, VIT_04s0069g00720, VIT_04s0069g00730, VIT_04s0069g00740, VIT_04s0069g00280	Significant enrichment of the functional category “Transport overview.Channels and pores.a-Type channels”.	✓
**LG 7a: 72.1–91.2 cM = 8,232-3,723,034bp (437 gene predictions**)		
IAA16: VIT_07s0141g00290*	Involved in auxin signalling; induced at pre-véraison both in HFPs and LFPs.	✓
Auxin-induced protein 22D: VIT_07s0141g00270*	Involved in auxin signalling; induced at pre-véraison both in HFPs and LFPs.	✓
Isopentenyltransferase 5: VIT_07s0104g00270*	Involved in cytokinin signalling; induced at pre-véraison both in HFPs and LFPs.	✓
Phytochrome A-associated F-box protein: VIT_07s0005g00340*	Involved in light signalling; induced at pre-véraison in HFPs.	✓
Myb domain protein 12: VIT_07s0005g01210 (*VvMYBF1*)	Involved in the regulation of flavonol biosynthesis (Czemmel *et al.*, 2009).	✓
BZIP DNA-binding protein BZIP53: VIT_07s0005g01450#	Involved in the regulation of flavonoid pathway in grapevine (Malacarne *et al*., upublished).	✓
Transparent testa 8 TT8: VIT_07s0104g00090	BrTT8 is involved in controlling the late biosynthetic genes of the flavonoid pathway (Li *et al.*, 2012); higher expression in skin than in flesh (Lijavetzky *et al.*, 2012).	✓
**LG 11b: 0–6.6 cM = 16,412,651-19,815,971bp (202 gene predictions**)		
ABC protein 3 ATNAP3 non-intrinsic: VIT_11s0052g00540*	Induced at pre-véraison both in HFPs and LFPs; higher expression in skin than in flesh (Lijavetzky *et al.*, 2012).	✓
UDP-glucose flavonoid 3-O-glucosyltransferase: VIT_11s0052g01600, VIT_11s0052g01630	*VvGT6* and *VvGT5* contribute to the chemical diversity of flavonol glycosides (Ono *et al.*, 2010). VIT_11s0052g01630 co-expressed with *VvFLS4*.	✓
**LG 14: 0–17.5 cM = 24,431,173-30,252,880bp (656 gene predictions**)		
MLK/Raf-related protein kinase 1: VIT_14s0066g01400*	Induced at pre-véraison both in HFPs and LFPs (higher fold change in HFPs); higher expression in skin than in flesh (Lijavetzky *et al.*, 2012).	✓
UVH3 (ultraviolet hypersensitive 3): VIT_14s0066g01630*	Induced at pre-véraison both in HFPs and LFPs (higher fold change in HFPs).	✓
Myb domain protein 24 (*VvMyb24*):: VIT_14s0066g01090	Transcript up-regulated by UV-radiation in Tempranillo berries (Carbonell-Bejerano *et al.*, 2014).	✓
Chalcone synthase: VIT_14s0068g00920*, VIT_14s0068g00930	VIT_14s0068g00920 induced at maturity both in HFPs and LFPs (higher fold-change in HFPs); higher expression in skin than in flesh (Lijavetzky *et al.*, 2012); co-expressed with *VvFLS4*.	✓
**LG 15: 38.4–49.3 cM = 99,408 -10,893,099bp (367 gene predictions**)		
Glutathione S-transferase 8 (*GSTU8*): VIT_15s0024g01540, VIT_15s0024g01630, VIT_15s0024g01650, VIT_15s0107g00150, VIT_15s0107g00160	GSTs have been associated to flavonoids’ transport (Dixon *et al.*, 2010).	✓
**LG 16: 0.0–12.2 cM = 19,046,519 -22,016,042bp (290 gene predictions**)		
Flavanone 3-hydroxylase: VIT_16s0098g00860*	Induced at maturity both in HFPs and in LFPs (higher fold-change in LFPs); higher expression in skin than in flesh (Lijavetzky *et al.*, 2012); co-expressed with genes of “flavonoid biosynthetic process”.	
Caffeic acid O-methyltransferase: VIT_16s0098g00850*	Induced at maturity in HFPs.	✓
**LG 18a: 0.0–36.1 cM = 4,354-7,861,866bp (740 gene predictions**)		
Purine permease 1 (*PUP1*): VIT_18s0001g06940*, VIT_18s0001g06950*, VIT_18s0001g06300, VIT_18s0001g06910	VIT_18s0001g06940 and VIT_18s0001g06950 induced at pre-véraison both in HFPs and LFPs; VIT_18s0001g06940 higher expression in skin than in flesh (Lijavetzky *et al.*, 2012); involved in “cytokinin metabolism” found as significantly enriched in the QTL region.	✓
Cis-zeatin O-beta-D-glucosyltransferase VIT_18s0001g05910*, VIT_18s0001g05950, VIT_18s0001g06090, VIT_18s0001g06120	VIT_18s0001g05910* induced in green versus ripe berries in both HFPs and LFPs. Significant enrichment of the functional category “Signalling.Hormone Signalling.Cytokinin Signalling”.	✓
ER6 protein universal stress protein (USP) family: VIT_18s0001g07360*	Involved in ethylene signalling; induced in green versus ripe berries in HFPs.	✓
Ethylene-inducible CTR1: VIT_18s0001g07700*	Involved in ethylene signalling. Induced in green versus ripe berries in HFPs and more expressed in the skin than in the flesh (Lijavetzky *et al.*, 2012).	✓
Flavonol synthase: VIT_18s0001g03470*(*VvFLS4*)	Induced at maturity in HFPs; expressed in the skin at early stage and during ripening (Fujita *et al.*, 2006); up-regulated by UV-radiation in Tempranillo ripe berries (Carbonell-Bejerano *et al.*, 2014).	
Sinapyl alcohol dehydrogenase: VIT_18s0122g00450 (*VvCAD6*)	Up-regulated by UV-radiation in Tempranillo ripe berries (Carbonell-Bejerano *et al.*, 2014).	
Nitrate reductase 2 (*NR2*): VIT_18s0001g03910	Induced in green versus ripe berries in LFPs; co-expressed with *VvGT5* and *VvGT6.*	✓
LIM domain protein WLIM: VIT_18s0001g09040*	Induced in green versus ripe berries in in HFPs and LFPs; co-expressed with *VvMYBF1.*	✓

The symbols * and # indicate genes found to be differentially expressed or not in this study.

Of the flavonol-specific QTLs, only a few regulated a specific trait: QTLs on LGs 4a and 14 controlling the 3′Meth/3′OH ratio (only on the white progeny), and a QTL on LG 18a controlling the Kaemp/Que+Isor ratio. All other QTL displayed a pleiotropic effect, as suggested by their influence on many traits visually highlighted in Fig. 2. Interestingly, the region on LG 7a associated with Kaemp (explaining 14% of total variance) co-localizes with the locus of *VvMYBF1*, whose expression shows a peak at early berry development (Czemmel *et al.*, 2009), in agreement with the profile of the ‘early peaking’ flavonols such as Kaemp (Sternad Lemut *et al.*, 2013). All the regions specifically related to flavonol content represent a valuable resource to search for new candidate genes potentially involved in the fine regulation of flavonols in the mature berry.

Except for the major QTL on LG 2, the other regions shared with anthocyanins were related to more general activities. For example, the QTL on LG 1a primarily regulates the methylation level (23–35% of explained variance), the QTL on LG 6 the hydroxylation level (28% of explained variance), and the QTL on LG 10 the 5′-methylation level (10% of explained variance).

### Gene expression analysis

Of the coloured offspring, four HFPs and four LFPs were selected, and compared at the transcriptomic level by means of a custom Affymetrix GeneChip using an approach based on bulked segregant gene expression analysis.

Probe sets with significant differential expression (DEPs) between LFPs and HFPs were selected in a two-step analysis and under the assumption that individuals of the LFP and HFP groups can be treated as four biological replicates (pseudoreplicates). Since the expression levels reflect the average of each pool and expression outliers have reduced effect, genes showing differential expression between the two groups can be associated with the trait under investigation. This type of approach has been successfully used in previous works (Guo *et al.*, 2006; Fernandez-Del-Carmen *et al.*, 2007; Chen *et al.*, 2010, 2011; Kloosterman *et al.*, 2010; Pons *et al.*, 2014).

Comparative analysis of the transcriptome at three developmental stages (PV versus VER, VER versus MAT, and PV versus MAT) within each bulk revealed the differential expression of 4834 and 3930 probe sets in HFPs and in LFPs, respectively. Of these, 1579 and 675 were exclusively modulated in HFPs and LFPs, while 3109 of 3255 were modulated in both, but with a difference in FC >50% (Supplementary Table S4 at *JXB* online).

**Fig. 3.**
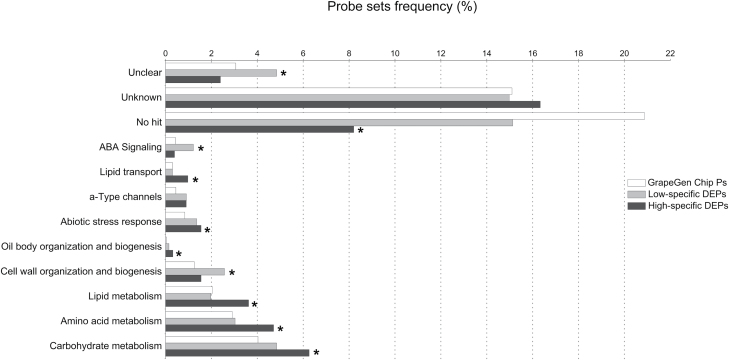
Transcriptomic analysis of high- and low-flavonol producers (HFPs and LFPs) by Affymetrix GrapeGen Chip. Assignment of the probe sets exclusively modulated in HFPs (dark grey) and in LFPs (light grey) to 84 functional categories of the third level of definition from the custom-made catalogue based on plant-related terms from the GO vocabulary and MIPS FunCat (Grimplet *et al.*, 2012). Only the functional categories significantly enriched in HFP or LFP data sets (black and grey, respectively) compared with the GrapeGen Chip (white) according to a hypergeometric test (*P*<0.05) are shown.

Differentially expressed probe sets were assigned to 84 functional categories at the third level of definition taken from the annotation of the GeneChip probe sets (Ashburner *et al.*, 2000; Ruepp *et al.*, 2004). Although the group of probe sets that could not be associated with any biological process (‘unknown’, ‘unclear’, and ‘unclassified’) or that could not find any significant hit (‘no hit’) was the largest in the lists of both the HFP and the LFP DEPs, 10 categories differently represented when compared with the GeneChip categories were identified (Fig. 3). In the HFP data set, the categories of the cellular process ‘Oil body organization and biogenesis’, of the primary metabolism ‘Amino acid metabolism’, ‘Carbohydrate metabolism’, ‘Lipid metabolism’, of the response to stress ‘Abiotic stress response’, and of transport ‘a-Type channels’ and ‘Lipid transport’ were significantly over-represented, while the ‘no hit’ category was somewhat surprisingly under-represented (Fig. 3). Noteworthy is the case of the 24 probe sets of the ‘Abiotic stress response’ category corresponding to genes mainly encoding dehydration- and light stress-responsive proteins, known to affect the flavonol profiles in the grape berry skin (Flamini *et al.*, 2013; Teixeira *et al.*, 2013): three abscisic acid (ABA)-responsive dehydration-responsive proteins 22 (Yamaguchi-Shinozaki and Shinozaki, 1993; Hanana *et al.*, 2008) and one ELIP1 (early light-inducible protein) were identified whose activity in *Arabidopsis* was related to the presence of photoprotective flavonoids (Heddad *et al.*, 2006). On the other hand, the LFP data set was significantly and exclusively enriched in two categories, ‘Cell wall organization and biogenesis’ and ‘ABA signalling’. The large fraction of differentially expressed probe sets, corresponding to genes with unknown function potentially relevant for the fine control of the flavonol pathway, highlights that knowledge of the process is still scarce and is a valuable source for further investigation. The differential expression between HFPs and LFPs was used as the main criterion to support the choice of candidates within QTLs for the genes represented on the GeneChip.

### Candidate gene identification

The number of genes within each QTL confidence interval varied from a minimum of 101 (LG 18b) to a maximum of 740 (LG 18a) (Fig. 2). The criteria adopted for candidate gene selection are detailed in the Materials and methods. However, functional annotation deriving from previous knowledge present in the literature was particularly considered, in the case of transcription regulators and structural enzymes related to phenylalanine, phenylpropanoid, flavonoid, and flavonol metabolism, as well as transport and signalling/response to stimulus. The selected candidate genes are listed in Table 1 and Supplementary Table S7 at *JXB* online, together with functional evidence from this study or previous studies.

Among the selected genes, special attention was paid to those encoding specific enzymes of the flavonol metabolism pathway as well as to fine regulators of the trait, which represent novel candidates identified in this study (Table 1).

Glycosylation is an important step in flavonol biosynthesis, enhancing their water stability and accumulation in plant cells. This process is regio-specific and is driven by specific glycosyltransferases (UGTs) for each flavonoid class (Ono *et al.*, 2010). Two previously characterized flavonol UGTs (VIT_11s0052g01630=*VvGT5* and VIT_11s0052g01600=*VvGT6*) are located within a QTL on LG 11 (11b) that was found to be specific for flavonol biosynthesis.

The conversion of Myr into Lar and Syr by 3′ and 5′ methylation is driven by the *O*-methyltransferases (OMTs) that convert delphinidin into petunidin and malvidin (LG 1; Hugueney *et al.*, 2009; Fournier-Level *et al.*, 2011; Costantini *et al.*, 2015). However two new putative caffeoyl-CoA OMTs for the methylation of Myr to Syr (VIT_11s0016g02600 and VIT_11s0016g02610; *VvCCOAMT* genes in the present work) were identified in this study on LG 11a. These two genes are very similar to AT4G26220, which encodes an enzyme preferentially methylating the para position of flavanones and dihydroflavonols (Wils *et al.*, 2013). Considering hydroxylation, it was confirmed that two of the five flavonoid 3′,5′-hydroxylases identified as candidates within the QTL on LG 6 are involved in the hydroxylation of both anthocyanins and flavonols. This is in agreement with the significant correlation between the triOH/diOH ratio in the two classes observed in the ‘Syrah’×’Pinot Noir’ progeny (*r*=0.55; Supplementary Table S2 at *JXB* online) and in the grapevine germplasm (*r*=0.45; Mattivi *et al.*, 2006)).

Three diphenol oxidases (VIT_18s0164g00090, VIT_18s0164g00110, and VIT_18s0164g00170; *VvLAC14* in the present work) and the putative homologue of AtPrx12 (VIT_18s0072g00160; *VvPrx12* in the present work) (QTL on LG 18b, common to anthocyanins) were considered as potentially involved in the oxidative turnover of flavonols among flavonoids (Pourcel *et al.*, 2005; Oren-Shamir, 2009; Zipor *et al.*, 2014). Genes playing a role in flavonoid transport in many plants including grapevine (Zhao and Dixon, 2010) were also listed among the best candidates for flavonol sequestration in the vacuole and the cell wall: priority was given to glutathione S-transferases on LGs 1a, 4a, and 15 (Dixon *et al.*, 2010) and glutamate receptor proteins (LG 4), whose relationship with flavonoids is due to their neuroprotective effect under stress (Nakayama *et al.*, 2011).

At the same time, genes encoding regulatory factors or proteins whose expression is known to be modulated by external or endogenous factors are identified.

Flavonol accumulation has been shown to be highly influenced by UV-B light and auxin and ethylene signalling, due to the important role played by flavonols as UV protectants during berry ripening (reviewed by Flamini *et al.*, 2013; Teixeira *et al.*, 2013) and as modulators of auxin transport (Lewis *et al.*, 2011). It is worth noting that most of them are located within QTLs specifically involved in flavonol variation. This is true in the case of VIT_07s0005g01210 (*VvMYBF1*), VIT_14s0066g01090 (*MYB24*), VIT_17s0000g06930 (*VvUNE10* in the present work), VIT_18s0122g00450 (*VvCAD6* in the present work), and VIT_18s0001g03470 (*VvFLS4*) as these transcripts were found to be up-regulated by UV-radiation in the skin of ripe berries of the cultivar Tempranillo (Carbonell-Bejerano *et al.*, 2014). VvFLS4 and VvFLS5 are the only two isoforms of flavonol synthase that accumulate in the skin of berries at the ripening stage (Fujita *et al.*, 2006). As potential candidate genes, attention was also focused on the bZIP family members falling within the identified QTL regions, since they are known to be part of light-mediated regulation of some flavonoid biosynthetic genes (Hartmann *et al.*, 2005; Czemmel *et al.*, 2009). VIT_05s0020g01090 (*VvbZIP15* in Liu *et al.*, 2014) is the putative homologue of *AtHY5*, which regulates PFG1/MYB112, a flavonol-specific activator of flavonoid biosynthesis in response to light (Stracke *et al.*, 2010). One bZIP gene probably involved in flavonol metabolism is VIT_07s0005g01450 (*VvbZIP22* in Liu *et al.*, 2014), in proximity to *VvMYBF1* within a QTL on LG 7 and whose expression during berry development and upon UV-light treatment correlates well with flavonol accumulation (G. Malacarne *et al*., unpublished). A very similar bZIP in terms of sequence and of expression profile in HFPs and LFPs is VIT_05s0077g01140 (*VvbZIP14* in Liu *et al.*, 2014) which was found in a QTL on LG 5. Both sequences are highly similar to that of *AtbZIP53*, coding for a bZIP related to amino acid metabolism during plant starvation (Dietrich *et al.*, 2011).

Three genes coding for a proton-dependent oligopeptide transport family protein (VIT_17s0000g05550), for the putative homologue of AtMYB66 (VIT_17s0000g08480), and for a chlorophyll a-b binding protein 4 (VIT_17s0000g06350) were localized within the QTL on LG 17 which appeared to be associated with different metabolic traits. These genes were co-expressed with *VvMYBF1* during berry development (Fasoli *et al.*, 2012; Lijavetzky *et al.*, 2012; Dal Santo *et al.*, 2013) and they are differentially expressed in HFPs versus LFPs.

Finally, genes such as VIT_01s0150g00300 and VIT_01s 0026g02620, VIT_07s0141g00290 and VIT_07s0141g00270, VIT_11s0016g03640 and VIT_12s0059g00190, although located within different QTL intervals, all appear to be involved in auxin-mediated flavonoid regulation of plant growth and development (Lewis *et al.*, 2011), or during response to UV-B radiation (Berli *et al.*, 2010). There is indeed evidence of flavonols influencing auxin transport and auxin-dependent physiological processes (Buer and Muday, 2004), as well as auxin and ethylene tuning flavonol accumulation (Lewis *et al.*, 2011). Moreover, factors that induce flavonol biosynthesis such as the light levels also affect auxin transport (Buer *et al.*, 2006).

### Conclusions

In this work, an integrative approach based on transcriptional profiling and metabolic QTL analyses was adopted to examine the regulation of berry flavonol content and composition in wine cultivars at the ripe berry stage. The seven QTL regions associated exclusively with flavonol biosynthesis represent a valuable resource for breeding and selection of new high quality cultivars.

## Supplementary data

Supplementary data are available at JXB online.

Supplementarty Text.

Figure S1. (A) Comparison of microarray and real-time RT–PCR analyses. (B) Consistency of expression tested among replicates for *VvMYBF1* and *VvFLS4* genes by real-time RT–PCR.

Table S1. Flavonol variation in the white and coloured individuals of the ‘Syrah’x‘Pinot Noir’ progeny.

Table S2. Correlations between metabolites in the coloured progeny.

Table S3. QTLs for flavonol content and composition identified in the ‘Syrah’x‘Pinot Noir’ progeny.

Table S4. GrapeGen Chip probe sets differentially expressed during development in high- and low-flavonol producers.

Table S5. Primers used in quantitative RT-PCR analysis for microarray validation.

Table S6. Test for over-representation of specific functional categories in QTL regions.

Table S7. Genes with a potential role in the regulation of both flavonol and anthocyanin content and composition.

Text S1. Validation of microarray results by real-time RT–PCR analysis.

Supplementary Data
