# Supplementary material for: Regulation of flavonol content and composition in (Syrah×Pinot Noir) mature grapes: integration of transcriptional profiling and metabolic quantitative trait locus analyses
Source: J Exp Bot. 2015 Jun 12;66(15):4441–53. doi: 10.1093/jxb/erv243 (PMC4507773; doi:10.1093/jxb/erv243)
Supplement: Supplementary Data [file supp_66_15_4441__index.html]

Regulation of flavonol content and composition in (Syrah×Pinot Noir) mature grapes: integration of transcriptional profiling and metabolic quantitative trait locus analyses — Regulation of flavonol content and composition in (Syrah×Pinot Noir) mature grapes: integration of transcriptional profiling and metabolic quantitative trait locus analyses — Regulation of flavonol content and composition in (Syrah×Pinot Noir) mature grapes: integration of transcriptional profiling and metabolic quantitative trait locus analyses — Regulation of flavonol content and composition in (Syrah×Pinot Noir) mature grapes: integration of transcriptional profiling and metabolic quantitative trait locus analyses — Supplementary Data 

# Regulation of flavonol content and composition in (Syrah×Pinot Noir) mature grapes: integration of transcriptional profiling and metabolic quantitative trait locus analyses

## Supplementary Data

Data files

**Files in this Data Supplement:**

- Supplementary Data - Supplementary Data
- Supplementary Data - Supplementary Data
