# Supplementary material for: Regulation of flavonol content and composition in (Syrah×Pinot Noir) mature grapes: integration of transcriptional profiling and metabolic quantitative trait locus analyses
Source: J Exp Bot. 2015 Jun 12;66(15):4441–53. doi: 10.1093/jxb/erv243 (PMC4507773; doi:10.1093/jxb/erv243)
Supplement: Supplementary Data [file supp_erv243_jexbot144105_file002.pdf]

Journal of Experimental Botany

Regulation of flavonol content and composition in (Syrah x Pinot Noir) mature grapes: integration of transcriptional profiling and metabolic QTL analyses.

Malacarne, G., Costantini, L. , Coller, E. , Battilana, J., Velasco, R. , Vrhovsek, U. , Grando, M.S., Moser, C.

Table S1. Range of variation of each flavonol -(expressed as mg/kg of berry) in white and colored individuals of the Syrah x Pinot Noir progeny in four seasons. Minimum values were considered different from zero when they exceeded the limits of detection and quantification of each compound. Abbreviations: Kaemp=Kaempferol; Que=Quercetin; Isor=Isoramnetin; Myr=Myricetin; Lar=Laricitrin; Syr=Syringetin;Other abbreviations: Tot_Flav= total flavonols; Min = minimum; Max = maximum.

**Metabolite**	**2007**		**2008**		**2009**		**2011**	
	**Min**	**Max**	**Min**	**Max**	**Min**	**Max**	**Min**	**Max**
**White-Skinned (WS) F1 individuals**								
Kaemp	1.7	21.5	0.7	9.2	0.0	7.2	0.0	16.2
Que	9.3	57.2	7.5	50.7	1.0	30.1	3.6	40.6
Isor	0.0	1.2	0.0	0.8	0.0	1.0	0.0	1.2
Myr	0.0	0.0	0.0	0.0	0.0	0.0	0.0	0.0
Lar	0.0	0.0	0.0	0.0	0.0	0.0	0.0	0.0
Syr	0.0	0.0	0.0	0.0	0.0	0.0	0.0	0.0
Tot_Flav	11.5	75.9	8.8	59.5	1.0	37.5	4.1	51.3
**Red-Skinned (RS) F1 individuals**								
Kaemp	0.0	7.5	0.0	7.4	0.0	4.6	0.3	8.4
Que	2.4	59.8	3.7	61.0	0.9	49.7	7.4	55.0
Isor	0.7	15.5	0.0	10.0	0.0	6.9	0.2	12.9
Myr	1.3	56.5	0.0	64.3	1.9	40.5	0.0	5.4
Lar	0.0	8.7	0.0	5.9	0.0	5.8	0.0	11.7
Syr	0.0	6.3	0.0	4.1	0.0	4.6	0.0	5.0
Tot_Flav	5.3	125.7	9.6	141.8	4.6	86.8	12.8	82.2

Table S2. Spearman rank-order correlations between flavonols (A), between flavonols and anthocyanins (B) and between flavonol and anthocyanin ratios (C) in the colored progeny. Correlations coefficients are average values from four seasons (the range of variation is reported in brackets). Numbers in apex indicate the years with significant correlation (only when<4 years). Different colors are according to a colored scale from black (r=1) to white (r=0).

Abbreviations: Kaemp=Kaempferol; Que=Quercetin; Isor=Isoramnetin; Myr=Myricetin; Lar=Laricitrin; Syr=Syringetin; Cya=cyanidin, Peo=Peonidin, Delph=Delphinidin, Pet=Petunidin, Malv=Malvidin, derivatives (3-monoglucoside + 3-monoglucoside-acetate + 3-monoglucoside-*p*-coumarate); triOH/diOH Flav= tri-hydroxylated (myricetin + laricitrin + syringetin)/ di-hydroxylated (quercetin+isoramnetin); triOH/diOH Anth= tri-hydroxylated (delphinidin + petunidin + malvidin 3-monoglucoside)/ di-hydroxylated (cyanidin + peonidin 3-monoglucoside); 3'Meth/3'OH Flav= isoramnetin/quercetin; 3'5'Meth/3'5'OH Flav= syringetin/myricetin; 3'Meth/3'OH Anth= peonidin 3-monoglucoside/cyanidin 3-monoglucoside; 3'5'Meth/3'5'OH Anth= malvidin 3-monoglucoside/delphinidin 3-monoglucoside (data on anthocyanins are from (Costantini *et al.*, 2015)).

**A**	**Kaemp**	**Que**	**Isor**	**Myr**	**Lar**	**Syr**
**Kaemp**						
**Que**	0.84 (0.82/0.88)					
**Isor**	0.66 (0.60/0.71)	0.73 (0.64/0.77)				
**Myr**	0.37 (0.34/0.41)	0.51 (0.46/0.59)	0.26 (018/0.35)			
**Lar**	0.49 (0.46/0.53)	0.55 (0.47/0.69)	0.52 (0.46/0.61)	0.78 (0.68/0.85)		
**Syr**	0.27 (0.20/0.36)^{3}	0.34 (0.23/0.45)^{2}	0.54 (0.48/0.62)^{3}	0.55 (0.45/0.62)	0.76 (0.57/0.88)	
**B**						
**Cya derivatives**	0.21 (0.18/0.25)^{2}	0.38 (0.33/0.48)	0.31^{1}	0.40 (0.35/0.42)	0.28 (0.22/0.33)	NS
**Peo derivatives**	0.21 (0.13/0.28)^{3}	0.36 (0.28/0.45)	0.40 (0.35/0.46)	0.20^{1}	0.18 (0.10/0.24)^{2}	0.20^{1}
**Delph derivatives**	NS	0.28 (0.21/0.30)	NS	0.64 (0.63/0.65)	0.42 (0.36/0.53)	0.25 (0.10/0.6)^{2}
**Pet derivatives**	NS	0.38 (0.34/0.42)^{2}	NS	0.65 (0.63/0.67)	0.45 (0.37/0.53)	0.29 (0.11/0.62)^{2}
**Malv derivatives**	NS	0.20 (0.12/0.24)^{2}	NS	0.60 (0.50/0.7)	0.52 (0.41/0.62)	0.51 (0.31/0.74)

C	TriOH/diOH Anth		
TriOH/diOH Flav	0.55 (0.48/0.62)	3’5’Meth/3’5’OH Anth	
3’5’Meth/3’5’OH Flav		0.6 (0.45/0.70)	3’Meth/3’OH Anth
3’Meth/3’OH Flav			0.49 (0.28/0.63)

Correlations are significant at the 0.01 level.

Table S7. Genes with a potential role in the regulation of flavonol and anthocyanin content and composition. The symbols * and # indicate genes found to be differentially expressed or not in this study.

Candidate gene	Functional evidence	This study	Common to anthocyanins
**LG 1a: 0.0-18.8 cM = 10,284,104-23,021,414 bp (636 gene predictions)**			
Caffeoyl-CoA O-methyltransferase: VIT_01s0010g03460, VIT_01s0010g03470 (*VvOMT3*), VIT_01s0010g03490 (*VvOMT2*), VIT_01s0010g03510* (*VvOMT1*)	VvOMT1 highly induced at véraison in HFPs and in LFPs; more expressed in the skin than in the flesh (Lijavetzky *et al.*, 2012).	✓	✓
Glutathione S-transferase: VIT_01s0026g01340#, VIT_01s0026g01370#, VIT_01s0026g01380#, VIT_01s0026g02390#, VIT_01s0026g02370#, VIT_01s0026g02400*	AtGSTU17 and ZmBz2 co-expressed with the flavonoid biosynthetic genes (Marrs *et al.*, 1995; Yonekura-Sakakibara *et al.*, 2008); VIT_01s0026g01340 more expressed in the skin than in the flesh; VIT_01s0026g02400 induced at maturity both in HFPs and LFPs, with a slightly higher fold change in HFPs; VIT_01s0026g01340 more expressed in the skin than in the flesh (Lijavetzky *et al.*, 2012); significant enrichment of the functional category "Metabolism.Primary metabolism.Coenzyme and prosthetic group metabolism".	✓	✓
Indole-3-acetic acid amido synthetase: VIT_01s0150g00300	Involved in auxin signaling; co-expressed with *VvMYBF1*; flavonol accumulation may be induced by both auxin and ethylene and have implicated these specialized metabolites in hormone-dependent developmental pathways (Lewis *et al.*, 2011).	✓	
Expansin: VIT_01s0026g02620*	Induced at véraison both in HFPs and LFPs, with a higher fold change in HFPs; more expressed in the skin than in the flesh (Lijavetzky *et al.*, 2012); co-expressed with genes significantly enriched in "flavonol metabolic process" category; involved in auxin signalling.	✓	
Glutaredoxin: VIT_01s0010g01680*	Induced at véraison both in HFPs and LFPs, with a higher fold change in HFPs; more expressed in the skin than in the flesh (Lijavetzky *et al.*, 2012); co-expressed with genes significantly enriched in "flavonol metabolic process" category; involved in auxin	✓	

	signalling.		
Calmodulin binding protein:<br>VIT_01s0026g01790*	Induced at véraison both in HFPs and LFPs;<br>co-expressed with genes significantly enriched in "regulation of phenylpropanoid metabolic process" category.<br>Induced at maturity in HFPs	✓	
Jasmonate ZIM domain-containing protein 10:<br>VIT_01s0146g00480*	Induced at maturity in HFPs.	✓	
Jasmonate ZIM domain-containing protein 10:<br>VIT_01s0146g00480*<br>WRKY DNA-binding protein 75:<br>VIT_01s0010g03930	More expressed in the skin than in the flesh (Lijavetzky *et al.*, 2012); co-expressed with genes significantly enriched in "response to biotic stimulus" category	✓	
**LG 2: 45.3-49.5 cM = 14,047,004-18,251,419 bp (149 gene predictions)**			
MYB domain protein: VIT_02s0033g00370 (*VvMYBA4*), VIT_02s0033g00380, VIT_02s0033g00390* (*VvMYBA2*), VIT_02s0033g00410* (*VvMYBA1*), VIT_02s0033g00430, VIT_02s0033g00440, VIT_02s0033g00450* (*VvMYBA3*)	*VvMYBA1*, *VvMYBA2* and *VvMYBA3* induced at véraison both in HFPs and LFPs; co-expressed.		✓
Alternative oxidase 1D: VIT_02s0033g01380*	Involved in ABA signaling, induced at maturity both in HFPs and LFPs, with a higher fold change in HFPs; more expressed in the skin than in the flesh (Lijavetzky *et al.*, 2012).	✓	
ABA 8'-hydroxylase CYP707A1:<br>VIT_02s0087g00710*	Induced at véraison both in HFPs and LFPs; more expressed in the skin than in the flesh (Lijavetzky *et al.*, 2012); involved in terpenoid metabolism.	✓	
**LG 5: 47.4-60.1 cM = 745,680-4,176,378 bp (373 gene predictions)**			
Pathogenesis protein 10 [Vitis vinifera]:<br>VIT_05s0077g01530*, VIT_05s0077g01560*,<br>VIT_05s0077g01580*	VIT_05s0077g01530, VIT_05s0077g01560 induced at maturity both in HFPs and LFPs, with a higher fold change in HFPs; VIT_05s0077g01580 induced at maturity in HFPs; all three genes are more expressed in the skin than in the flesh (Lijavetzky *et al.*, 2012); involved in the response to biotic stimulus.	✓	
Beta-1,3-glucanase: VIT_05s0077g01150*	Induced at véraison and maturity in HFPs; co-expressed with *VvMYBF1*.	✓	
BZIP transcription factor BZIP53:<br>VIT_05s0077g01140*	Induced at pre-véraison and at maturity in HFPs :the profile is similar to that obtained for *VIT_07s0005g01450*, a general regulator of flavonoid pathway (Malacarne *et al.*, unpublished).	✓	

BZIP protein HY5 (HY5): VIT_05s0020g01090#	AtHY5/HYH involved in light signaling. HY5 is able to activate *PFG1/MYB112*, a flavonol-specific activator of flavonoid biosynthesis during plant development (Stracke *et al.*, 2010); co-expressed with genes significantly enriched in "flavonol biosynthetic process".		
TRNA pseudouridine synthase: VIT_05s0020g00860	Co-expressed with genes significantly enriched in "methylation" functional category; expressed in skin starting from véraison (Fasoli et al., 2012)	✓	
**LG 6: 12.2-25.6 cM = 12,767,560-17,912,115 bp (265 gene predictions)**			
Flavonoid 3',5'-hydroxylase: VIT_06s0009g02830 (*F3´5´Hc*)*, VIT_06s0009g02920 (*F3´5´Hi*), VIT_06s0009g03000 (*F3´5´Hk*), VIT_06s0009g03010 (*F3´5´Hk*)*, VIT_06s0009g03110 (*F3´5´Hn*)	VIT_06s0009g02830 and VIT_06s0009g03010 induced at véraison both in HFPs and LFPs, with a slightly higher fold change in HFPs; VIT_06s0009g03010 more expressed in the skin than in the flesh; VIT_06s0009g02920, VIT_06s0009g03000 and VIT_06s0009g03110 co-expressed with *VvGT5* and *VvGT6* both contributing to the chemical diversity of flavonol glycosides (Ono *et al.*, 2010). The genes' names are from (Falginella *et al.*, 2010).	✓	✓
APK2A (protein kinase 2A): VIT_06s0009g01810	Induced at maturity in HFPs; expressed in skin starting from véraison (Fasoli *et al.*, 2012); involved in signaling calcium-dependent.		✓
**LG10: 7.8-30.7 cM = 2,342,131-9,015,334 bp (382 gene predictions)**			
Starch synthase protein: VIT_10s0003g02880*	Induced at pre-véraison both in HFPs and in LFPs, with a higher fold-change in HFPs.	✓	
Flavonol synthase: VIT_10s0003g02430*, VIT_10s0003g02450#	VIT_10s0003g02430 induced at pre-véraison both in HFPs and in LFPs.	✓	
RKF1 (receptor-like kinase in flowers 1): VIT_10s0003g01960*, VIT_10s0003g01900	VIT_10s0003g01960 induced at pre-véraison both in HFPs and in LFPs; VIT_10s0003g01900 more expressed in the skin than in the flesh (Lijavetzky et al., 2012). Induced at maturity in HFPs; co-expressed with *VvbZIP22* (VIT_07s0005g01450) involved in the regulation of flavonoid pathway in grapevine (Malacarne et al., unpublished data).	✓	
WRKY DNA-binding protein 65: VIT_10s0003g01600*	Co-expressed with *VvMYBF1* and *VvFLS4*	✓	
LHCII type I CAB-1: VIT_10s0003g02890 Jasmonate ZIM domain-containing protein 8: VIT_10s0003g03790*	Induced at maturity both in HFPs and in LFPs, with a higher fold-change in LFPs.	✓	✓

**LG 11a: 25.1-51.0 cM = 5,471,701-5,477,250 bp (342 gene predictions)**			
MSS1 (sugar transport protein 13): VIT_11s0016g03400*	Induced at maturity both in HFPs and in LFPs, with a higher fold-change in LFPs; more expressed in the skin than in the flesh (Lijavetzky *et al.*, 2012).	✓	
Rac-like GTP-binding protein ARAC7 (GTPase protein ROP9): VIT_11s0016g03640*	Induced in green versus ripe berries in HFPs and in LFPs; co-expressed with *VvMYBF1; AtRAC7/ROP9* is a modulator of auxin and abscisic acid signaling (Nibau *et al.*, 2013).	✓	
Caffeoyl-CoA O-methyltransferase: VIT_11s0016g02600, VIT_11s0016g02610 (*VvCCOAMTs*)	Homologues to *AT4G26220*, that shows a strong preference for methylating the para position of flavanones and dihydroflavonols (Wils *et al.*, 2013).	✓	
**LG 12a: 12-26.2 cM = 2,354,117-5,309,868 bp (259 gene predictions)**			
Serine/threonine-protein kinase NAK: VIT_12s0028g01970*	Induced at pre-véraison both in HFPs and LFPs.	✓	
GPRI1 (GOLDEN2 1): VIT_12s0028g03100*	Induced at pre-véraison both in HFPs and LFPs.	✓	
DNAJ heat shock N-terminal domain-containing protein: VIT_12s0028g01740*	Induced at pre-véraison both in HFPs and LFPs.	✓	
Basic helix-loop-helix (bHLH) family: VIT_12s0028g02350*	Induced at pre-véraison both in HFPs and LFPs.	✓	
HSL1 (HAESA-like 1): VIT_12s0028g03060*	Induced at pre-véraison both in HFPs and LFPs.	✓	
Squamosa promoter-binding protein 4 (SPL4): VIT_12s0028g03350*	Induced at maturity both in HFPs and LFPs, with a higher fold-change in HFPs.	✓	
Ankyrin repeat family: VIT_12s0059g00050*, VIT_12s0028g03920#, VIT_12s0028g03950, VIT_12s0028g03960, VIT_12s0028g03990, VIT_12s0028g04000, VIT_12s0028g04010, VIT_12s0028g04020, VIT_12s0028g04030, VIT_12s0028g04040, VIT_12s0059g00060, VIT_12s0059g00080, VIT_12s0059g00090, VIT_12s0059g00100, VIT_12s0059g00110, VIT_12s0059g00120, VIT_12s0059g00130, VIT_12s0059g00150	VIT_12s0059g00050 induced at pre-véraison both in HFPs and LFPs; VIT_12s0059g00050*,VIT_12s0028g04000, VIT_12s0028g04040, VIT_12s0059g00080, VIT_12s0059g00090, VIT_12s0059g00100, VIT_12s0059g00110, VIT_12s0059g00150 co-expressed genes; significant enrichment of the category "Transport overview.Accessory factors involved in transport.Auxiliary transport proteins".	✓	
Beta-expansin (EXPB3): VIT_12s0059g00190	Involved in auxin signaling. More expressed in the skin than in the flesh (Fasoli *et al.*, 2012).	✓	
**LG 17: 30.1-57.5 cM = 4,059,831-9,864,117 bp (440 gene predictions)**			
COBRA-like protein 4: VIT_17s0000g05050*	VIT_17s0000g05050 reduced at véraison only in LFPs; proposed as a regulator of proanthocyanidin total content (Carrier et al., 2013).		✓
Myb domain protein 94: VIT_17s0000g06190*	VIT_17s0000g06190 induced at véraison only		✓

	in HFPs; more expressed in the skin than in the flesh (Lijavetzky *et al.*, 2012). *ZmMYB31* over-expression leading to a large increase in flavonoid level through the up-regulation of flavonoid biosynthetic genes (Fornale *et al.*, 2010).		
Flavonoid 3'-hydroxylase: VIT_17s0000g07210*	Induced at véraison only in HFPs; more expressed in the skin than in the flesh (Lijavetzky *et al.*, 2012).		✓
EDS1 (Enhanced disease susceptibility 1): VIT_17s0000g07560*	Involved in jasmonate signaling, induced at maturity only in HFPs; more expressed in the skin than in the flesh (Lijavetzky *et al.*, 2012).		✓
Unfertilized embryo sac 10 UNE10: VIT_17s0000g06930 (*VvUNE10*)	Transcript up-regulated by UV-radiation in the skin of Tempranillo ripe berries (Carbonell-Bejerano *et al.*, 2014).		✓
Proton-dependent oligopeptide transport (POT) family protein: VIT_17s0000g05550*	Induced at pre-véraison both in HFPs and in LFPs; co-expressed with *VvMYBF1*.	✓	
ATMYB66/WER/WER1 (WEREWOLF 1): VIT_17s0000g08480	Co-expressed with *VvMYBF1*.	✓	
LHCA4 (Photosystem I light harvesting complex gene 4): VIT_17s0000g06350	Induced in green versus ripe berries in HFPs and in LFPs; co-expressed with *VvMYBF1*.	✓	
**LG 18b: 64.0-72.6 cM = 16,264,791-19,460,695 bp (101 gene predictions)**			
Light stress-responsive one-helix protein: VIT_18s0072g00110*	Involved in light response. Induced in green versus ripe berries both in HFPs and LFPs	✓	
Peroxidase 12-like: VIT_18s0072g00160 (*VvPrx12*)**	Induced at véraison only in HFPs, at maturity both in HFPs and LFPs, with a higher fold change in HFPs; more expressed in the skin than in the flesh (Lijavetzky *et al.*, 2012).		✓
Diphenol oxidase: VIT_18s0164g00090, VIT_18s0164g00110, VIT_18s0164g00170 (*VvLAC14*)	Potentially involved in the oxidative degradation of flavonoids (Pourcel *et al.*, 2005); VIT_18s0164g00170 expressed in the skin at véraison and middle ripening (Fasoli *et al.*, 2012).	✓	

Text S1. Validation of microarray results by real-time RT-PCR analysis.

To technically validate the microarray results, we analyzed by real-time RT-PCR the expression of 10 differentially expressed genes (Figure S1A). We selected genes with different expression profiles in two groups of four individuals of the cross significantly divergent for their content. Although the gene expression values revealed by the two techniques were not always the same, the expression profiles were in very good agreement for all the tested genes.

Furthermore, we evaluated the expression level of some flavonoid structural genes by real-time RT-PCR comparing the variability among different genotypes (HFPs and LFPs) treated as biological replicates (pseudoreplicates) (as in our array experiment) and the variability among three different biological replicates of the same genotype (as common rule in array experiments) (Figure S1B). Although the samples for the analysis were collected in two different seasons, we found that the range of variability calculated as standard error among different genotypes (samples collected in 2007) and among biological replicates of the same genotype (samples collected in 2011) was comparable between the two cases, or much higher in the second case. In particular, it is evident that the two groups of genotypes are significantly different in the expression level of *VvMYBF1* and *VvFLS4*, encoding for the known regulator and enzyme involved in the biosynthesis of flavonols, correlating with the different biochemical level at maturity.

Figure S1. A) Comparison of microarray and real-time RT-PCR results for 10 differentially expressed probesets (two High-specific, two Low-specific and six commonly modulated but with low correlation between the two groups). Expression profiles of transcription factor VvbZIP22 (*VVTU7177_at*) and α-type channel Acquaporin PIP2B (*VVTU29911_at*), of Zinc Knuckle protein (*VVTU8019_at*) and Major cherry allergen Pru av 1.0202 (*VVTU5508_at)*, of transcription factor MYB CCA1 (*VVTU15954_at)*, UDP-d-apiose/udp-d-xylose synthase 1 AXS1 (*VVTU2442_at*), Dihydroflavonol-4-reductase (*VVTU2594_at*), Dof zinc finger protein DOF4.6 (*VVTU31051_at*), Indole-3-acetate beta-glucosyltransferase (*VVTU9394_at*), and transcription factor WRKY33 (*VVTU13081_at*). Blue dotted lines represent the expression levels as assessed by microarray analysis and reported as means and standard errors of the four genotypes (pseudoreplicates) selected as Low- (left side) and High- (right side) Flavonol Producers at three developmental stages in 2007. Histograms represent the relative expression levels (fold-change relative to the average expression value of all the genotypes in all the stages) as assessed by real-time RT-PCR and reported as means and standard errors of the four genotypes (pseudoreplicates) at the three stages. **B)** Consistency of expression tested among replicates for *VvMYBF1* and *VvFLS4* genes by real-time RT-PCR. Histograms represent the expression levels as assessed by Real-time RT-PCR, reported as means

and standard errors of the four genotypes (pseudoreplicates) selected as Low- (white bar) and High- (black bar) Flavonol Producers at three developmental stages in 2007, and as means and standard errors of three biological replicates for each genotype at the three stages in 2011 (bar with light grey grid =LFPs, bar with black grid = HFPs, the genotypes are presented in the order described in the abbreviations). Asterisks indicate significant changes (p<0.05) in the comparison between LFPs and HFPs at each stage in each season, tested by a Student' s t test performed on the log 2 transformed data (as input in the test average values, calculated considering the entire group of production, were used).

Abbreviations (panel A and B): RMA = Robust Multi-array Average; NRQ = Normalized Relative Quantity; PV = pre-véraison; VER = véraison; MAT = maturity, 3BR = three biological replicates, LFPs= low flavonol producers= 64_{F1}, 256_{F1}, 260_{F1}, Pinot Noir , HFPs= 16_{F1}, 56_{F1}, 63_{F1}, 223_{F1}.

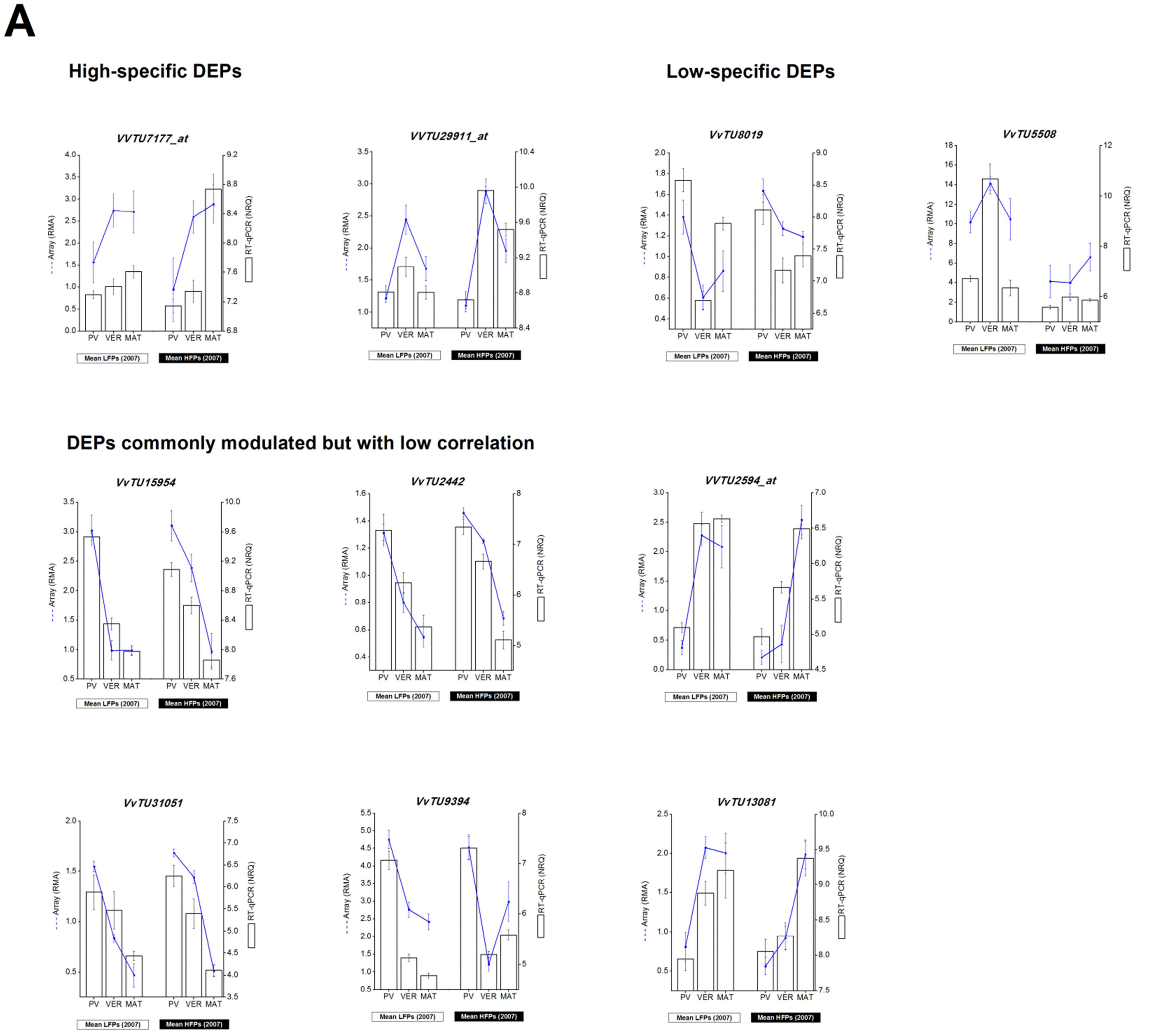

A
High-specific DEPs
Low-specific DEPs
VVTU7177_at
VVTU29911_at
VvTU8019
VvTU5508
DEPs commonly modulated but with low correlation
VvTU15954
VvTU2442
VVTU2594_at
VvTU31051
VvTU9394
VvTU13081
Array (RMA)
RT-qPCR (NRQ)
PV VER MAT
Mean LFPs (2007)
Mean HFPs (2007)

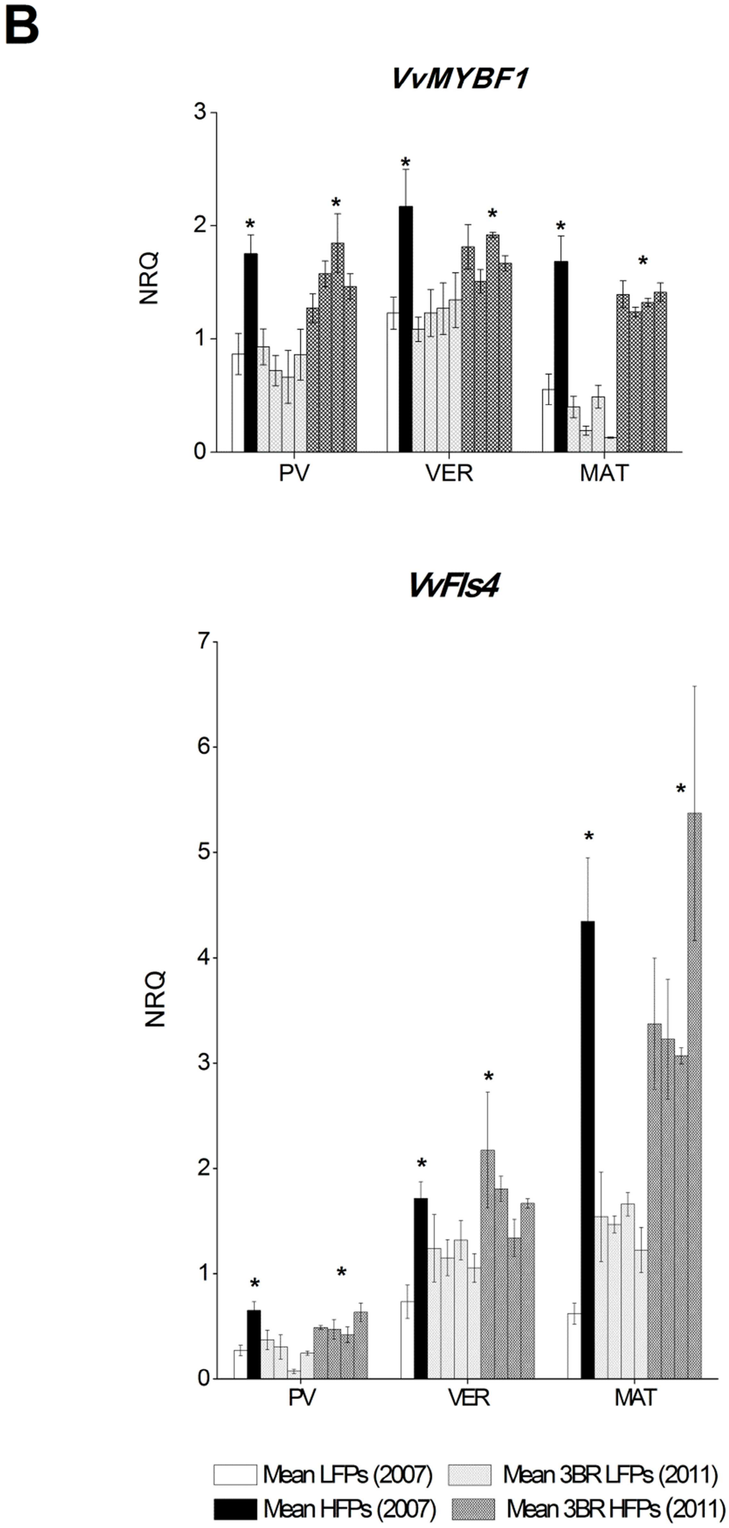
B
VvMYBF1
NRQ
PV
VER
MAT
VvFls4
Mean LFPs (2007)
Mean 3BR LFPs (2011)
Mean HFPs (2007)
Mean 3BR HFPs (2011)

References

Carbonell-Bejerano P, Diago MP, Martinez-Abaigar J, Martinez-Zapater JM, Tardaguila J, Nunez-Olivera E. 2014. Solar ultraviolet radiation is necessary to enhance grapevine fruit ripening transcriptional and phenolic responses. BMC Plant Biology **14**, 183.

Carrier G, Huang YF, Le Cunff L, Fournier-Level A, Vialet S, Souquet JM, Cheynier V, Terrier N, This P. 2013. Selection of candidate genes for grape proanthocyanidin pathway by an integrative approach. Plant Physiology Biochemistry **72**, 87-95.

Costantini L, Malacarne G, Lorenzi S, Troggio M, Mattivi F, Moser C, Grando MS. 2015. New candidate genes for the fine regulation of grapes' color. Journal of Experimental Botany **doi**: 10.1093/jxb/erv159.

Falginella L, Castellarin SD, Testolin R, Gambetta GA, Morgante M, Di Gaspero G. 2010. Expansion and subfunctionalisation of flavonoid 3',5'-hydroxylases in the grapevine lineage. BMC Genomics **11**, 562.

Fasoli M, Dal Santo S, Zenoni S, Tornielli GB, Farina L, Zamboni A, Porceddu A, Venturini L, Bicego M, Murino V, Ferrarini A, Delledonne M, Pezzotti M. 2012. The grapevine expression atlas reveals a deep transcriptome shift driving the entire plant into a maturation program. Plant Cell **24**, 3489-3505.

Fornale S, Shi X, Chai C, Encina A, Irar S, Capellades M, Fuguet E, Torres JL, Rovira P, Puigdomenech P, Rigau J, Grotewold E, Gray J, Caparros-Ruiz D. 2010. ZmMYB31 directly represses maize lignin genes and redirects the phenylpropanoid metabolic flux. *Plant J* **64**, 633-644.

Lewis DR, Ramirez MV, Miller ND, Vallabhaneni P, Ray WK, Helm RF, Winkel BS, Muday GK. 2011. Auxin and ethylene induce flavonol accumulation through distinct transcriptional networks. Plant Physiology **156**, 144-164.

Lijavetzky D, Carbonell-Bejerano P, Grimplet J, Bravo G, Flores P, Fenoll J, Hellin P, Oliveros JC, Martinez-Zapater JM. 2012. Berry flesh and skin ripening features in *Vitis vinifera* as assessed by transcriptional profiling. PLoS One **7**, e39547.

Marrs KA, Alfenito MR, Lloyd AM, Walbot V. 1995. A glutathione S-transferase involved in vacuolar transfer encoded by the maize gene Bronze-2. Nature **375**, 397-400.

Nibau C, Tao L, Levasseur K, Wu HM, Cheung AY. 2013. The Arabidopsis small GTPase AtRAC7/ROP9 is a modulator of auxin and abscisic acid signalling. Journal of Experimental Botany **64**, 3425-3437.

Ono E, Homma Y, Horikawa M, Kunikane-Doi S, Imai H, Takahashi S, Kawai Y, Ishiguro M, Fukui Y, Nakayama T. 2010. Functional Differentiation of the Glycosyltransferases That Contribute to the Chemical Diversity of Bioactive Flavonol Glycosides in Grapevines (Vitis vinifera). Plant Cell **22**, 2856-2871.

Pourcel L, Routaboul JM, Kerhoas L, Caboche M, Lepiniec L, Debeaujon I. 2005. TRANSPARENT TESTA10 encodes a laccase-like enzyme involved in oxidative polymerization of flavonoids in Arabidopsis seed coat. Plant Cell **17**, 2966-2980.

Stracke R, Favory JJ, Gruber H, Bartelniewoehner L, Bartels S, Binkert M, Funk M, Weisshaar B, Ulm R. 2010. The Arabidopsis bZIP transcription factor HY5 regulates expression of the PFG1/MYB12 gene in response to light and ultraviolet-B radiation. Plant Cell and Environment **33**, 88-103.

Wils CR, Brandt W, Manke K, Vogt T. 2013. A single amino acid determines position specificity of an *Arabidopsis thaliana* CCoAOMT-like O-methyltransferase. FEBS Letters **587**, 683-689.

Yonekura-Sakakibara K, Tohge T, Matsuda F, Nakabayashi R, Takayama H, Niida R, Watanabe-Takahashi A, Inoue E, Saito K. 2008. Comprehensive flavonol profiling and transcriptome coexpression analysis leading to decoding gene-metabolite correlations in Arabidopsis. Plant Cell **20**, 2160-2176.
